# Intake frequency of vegetables or seafoods negatively correlates with disease activity of rheumatoid arthritis

**DOI:** 10.1371/journal.pone.0228852

**Published:** 2020-02-13

**Authors:** Isao Murakami, Kosaku Murakami, Motomu Hashimoto, Masao Tanaka, Hiromu Ito, Takao Fujii, Mie Torii, Kaori Ikeda, Akiko Kuwabara, Kiyoshi Tanaka, Akiko Yoshida, Shuji Akizuki, Ran Nakashima, Hajime Yoshifuji, Koichiro Ohmura, Takashi Usui, Satoshi Morita, Tsuneyo Mimori

**Affiliations:** 1 Department of Rheumatology and Clinical Immunology, Graduate School of Medicine, Kyoto University, Kyoto, Japan; 2 Department of the Advanced Medicine for Rheumatic Diseases, Graduate School of Medicine, Kyoto University, Kyoto, Japan; 3 Department of Orthopedic Surgery, Graduate School of Medicine, Kyoto University, Kyoto, Japan; 4 Department of Rheumatology and Clinical Immunology, Wakayama Medical University, Wakayama, Japan; 5 Department of Human Health Sciences, Graduate School of Medicine, Kyoto University, Kyoto, Japan; 6 Department of Diabetes, Endocrinology, and Nutrition, Graduate School of Medicine, Kyoto University, Kyoto, Japan; 7 Department of Clinical Nutrition, Faculty of Comprehensive Rehabilitation, Osaka Prefecture University, Osaka, Japan; 8 Department of Faculty of Nutrition, Kobe Gakuin University, Hyogo, Japan; 9 Depatment of Biomedical Statistics and Bioinformatics, Kyoto University, Kyoto, Japan; University of Extremadura, SPAIN

## Abstract

**Objective:**

To clarify the relationship between dietary habit and disease activity of rheumatoid arthritis (RA).

**Methods:**

This study enrolled RA patients who met the ACR/EULAR 2010 classification criteria from Kyoto University Rheumatoid Arthritis Management Alliance (KURAMA) cohort in 2015. 22-item food frequency questionnaire (FFQ) was taken for the measurement of dietary habit in a single-institution cohort of RA (Kyoto University Rheumatoid Arthritis Management Alliance: KURAMA) in 2015. The disease activities of RA using the Disease Activity Score calculated based on the erythrocyte sedimentation rate (DAS28-ESR), Simplified Disease Activity Index (SDAI), Health Assessment Questionnaire (HAQ), and serum matrix metalloproteinase-3 (MMP-3) level, the use of disease-modifying anti-rheumatic drugs (DMARDs), disease duration, rheumatoid factor, anti-cyclic citrullinated antibody, and body mass index were also examined. All of them were combined and statistically analyzed.

**Results:**

441 RA patients (81% women; mean age 65 years; mean disease duration 15 years) were enrolled from the KURAMA cohort. Average Disease Activity Score-28 using the erythrocyte sedimentation rate (DAS28-ESR) was 2.7. Univariate analysis showed that intake frequency of vegetables had a statistically significant negative correlation with disease activity markers, such as DAS28-ESR (ρ = −0.11, *p*<0.01), Simplified Disease Activity Index (SDAI) (ρ = −0.16, *p*<0.001), matrix metalloproteinase-3 (MMP-3) (ρ = −0.21, *p*<0.0001), and Health Assessment Questionnaire (HAQ) (ρ = −0.13, *p*<0.01). Factor analysis with varimax rotation was done to simplify the relevance of disease activity to various food items. 22 foods were categorized into five dietary patterns: “seafoods”, “vegetables/fruits”, “meats/fried foods”, “snacks”, and “processed foods”. The multivariate analysis adjusted for clinically significant confounders showed that “seafoods” had statistically significant negative correlations with DAS28-ESR (β = −0.15, *p*<0.01), SDAI (β = −0.18, *p*<0.001), MMP-3 (β = −0.15, *p*<0.01), and HAQ (β = −0.24, *p*<0.0001). “Vegetables/fruits” had statistically significant negative correlations with SDAI (β = −0.11 *p*<0.05), MMP-3 (β = −0.12, *p*<0.01), and HAQ (β = −0.11, *p*<0.05)

**Conclusions:**

These results suggest that high intake frequency of vegetables/fruits and/or seafoods might correlate with low disease activity.

## Introduction

Rheumatoid arthritis (RA) is an autoimmune disease that mainly affects systemic joints [1]. Without appropriate treatment, persistent polyarthritis leads to joint deformity and functional disability in daily life. The precise etio-pathogenesis of RA is unclear, but the contribution of both genetic and environmental factors has been suggested. Various environmental factors, such as periodontal bacteria and smoking, are reported to be associated with RA in several epidemiological or basic medical studies [2], [3].

Dietary habits are important environmental factors which are associated with many diseases. However, the relationship between dietary habits and the pathogenesis or clinical course of RA has not been fully understood. In previous reports from Western countries, fish intake improves RA disease activity, whereas high consumption of fats and sugars worsens it [4]. Consumption of omega-3 polyunsaturated fatty acids improves disease activity of RA and JIA [5, 6]. The effectiveness of Nigella sativa oil in RA patients was also reported [7]. The Mediterranean diet may decrease arthralgia in RA patients [8].

However, in East Asian countries, dietary culture is much different from that in Western countries. Especially, there has been an increasing interest in the Japanese diet, which is rich in fishes and vegetables. Epidemiological studies focusing on the Japanese diet have been difficult because of the vast variation in Japanese foods [9], although the association between the Japanese diet and lower insulin resistance was reported [10]. There have been few reports in Japan on the association of dietary habits with disease activity and the numbers of patients in those reports were small [11, 12]. The purpose of this study is to clarify the relationship between dietary habits of RA patients and their disease activity.

## Patients and methods

### Patients

This study enrolled RA patients who met the ACR/EULAR 2010 classification criteria[13] from Kyoto University Rheumatoid Arthritis Management Alliance (KURAMA) cohort in 2015 [14, 15].

### Clinical parameters

The disease activities of RA using the Disease Activity Score calculated based on the erythrocyte sedimentation rate (DAS28-ESR)[16], Simplified Disease Activity Index (SDAI)[16], Health Assessment Questionnaire (HAQ)[17], the use of disease-modifying anti-rheumatic drugs (DMARDs), disease duration, rheumatoid factor, and anti-cyclic citrullinated antibody, and body mass index were examined. Serum matrix metalloproteinase-3 (MMP-3) level was measured by latex turbidimetrical immunoassay. The supplementary S1 File contains the detailed data on this study. This study was designed in accordance with the Helsinki Declaration. The ethics committee of Kyoto University Graduate School and Faculty of Medicine reviewed and approved the study protocol of the study. All the participants provided written informed consent to be enrolled in this cohort.

### Food frequency questionnaire

Food frequency questionnaire (FFQ) is useful for the detection of dietary habits [18, 19]. In this study, 22-item FFQ was took in order to clarify the correlation between dietary habits and RA disease activity. Most of the reported FFQs are too detailed to be answered in clinical settings. The FFQ in this study was a modified version of the FFQ which was reported previously [10]. This modified FFQ included 22 food items: meats, fishes, tofu (bean curd), egg, milk, vegetables, fruits, fried foods, cakes, juice, snacks, sweets, miso soup, Japanese-style pickles, ham, frozen foods, small fishes, canned tuna, squid, shellfishes, fish eggs, and fish pastes. In the questionnaire, the patients selected a choice from the list of their intake frequency for each food: 1 = less than once a month, 2 = once to three times a month, 3 = once or twice a week, 4 = three or four times a week, 5 = five or six times a week, 6 = once a day, 7 = twice a day, 8 = three times a day. The answers were checked by clinical staffs.

### Statistical analysis

The correlations between disease activity of RA and food intake frequency were statistically analyzed by univariate and multivariate analyses. First, Spearman’s rank correlation test was done to detect the correlation of the disease activity scores and each intake frequency of the 22 food items. Next, the factor analysis of the 22 food items was done to simplify the correlations among these food items and identify dietary patterns[20]. The 22 foods were then categorized into five dietary patterns using factor analysis with varimax rotation. Using the calculated factor scores, the correlations between food patterns and disease activity scores were conducted with a multiple regression analysis adjusted for the factors, which we thought as clinically significant, including age, disease duration, sex, body mass index, the dosage of methotrexate and prednisolone, biological disease-modifying anti-rheumatic drug use, and the titer of rheumatoid factor and anti-cyclic citrullinated peptide antibody. The statistical analyses in this study were performed with JMP Pro 13 software (SAS Institute Inc., Cary, NC, USA).

## Results

### Patients’ demographics

441 RA patients were enrolled. The patients’ demographics were as follows (Table 1). Mean age of the participants was 65 years old and 81% were female. Mean disease duration was 15 years and DAS28-ESR was 2.7 on average. MTX use was 66% and biologics use was 43%. Mean DAS28-ESR of the patients was 2.7±1.1 in 2016 and 2.7±1.1 in 2017.

**Table 1:** Rheumatoid arthritis patients’ backgrounds (n = 441).

Characteristic	Value
Age, years	65±13
Female sex, %	81
BMI, kg/m^2^	22±3.9
Disease duration, years	15±13
RA stage, median	3
RA class, median	2
Swollen joint count	0.75±1.6
Tender joint count	0.83±1.6
ESR, mm/h	22±17
CRP, mg/dL	0.42±1.0
MMP-3	98±94
Patient global assessment	26±24
Physician global assessment	8.9±11
DAS28-ESR	2.7±1.1
SDAI	5.5±5.7
ACPA positivity, %	72
RF positivity, %	74
HAQ-DI	0.63±0.70
Biologics use, %	43
MTX use, %	66
MTX use, mg/week	5.0±4.2
csDMARD (except MTX) use, %	37
Corticosteroid use, %	20
Prednisolone equivalent, mg/day	1.0±2.2

Results are expressed as the means ± SD or the percent (%) unless otherwise stated.

BMI: body mass index, ESR: erythrocyte sedimentation rate, CRP: C-reactive protein, MMP-3: matrix metalloproteinase-3, DAS28-ESR: disease activity score-28 calculated using the ESR, SDAI: Simplified Disease Activity Index, HAQ: Health Assessment Questionnaire, ACPA: anti-cyclic citrullinated peptide antibody, MTX: methotrexate, csDMARD: conventional synthetic disease modifying anti-rheumatic drug

### Intake frequency of the 22 foods

All the patients completely answered every item of the food frequency questionnaire (Table 2). For example, the intake frequencies for meats and fishes were comparable (median value was three or four times a week), and the participants tended to eat vegetables and fruits more than once a day.

**Table 2:** Frequency of each food.

Food	Frequency							
	<1/month	1-3/month	1-2/week	3-4/week	5-6/week	1/day	2/day	3/day
Meat	12	30	124	199	38	31	5	2
Fish	5	26	136	208	28	31	6	1
Tofu	5	34	144	131	58	54	13	2
Egg	9	27	138	132	55	73	2	5
Milk	94	23	34	47	30	177	24	12
Vegetable	3	6	9	57	62	128	106	70
Fruits	19	38	63	70	48	156	29	18
Fried food	16	94	209	96	12	12	1	1
Cake	25	103	150	82	21	55	4	1
Juice	118	77	88	50	26	61	9	12
Snack food	164	88	105	44	17	20	2	1
Sweets	53	96	115	85	32	49	5	6
Miso soup	22	52	119	99	63	78	3	5
Pickles	71	62	89	62	36	79	37	5
Ham	34	90	150	111	28	25	2	1
Frozen food	98	111	118	75	18	17	3	1
Small fish	47	126	152	59	20	32	4	1
Canned tuna	208	188	33	6	2	2	2	0
Squid	55	256	94	31	2	2	1	0
Shellfish	130	232	61	12	4	2	0	0
Roe	193	190	48	5	2	3	0	0
Fish paste	84	187	116	39	10	4	0	1

### Correlation between intake frequency and disease activity

Spearman’s rank correlation test was done to detect the correlation between the frequency of ingesting certain diets and the level of disease activity. This analysis showed that several food frequencies had statistically significant correlations with certain activity markers (Table 3). Especially, the intake frequency of vegetables had a statistically significant negative correlation with all activity markers—i.e., DAS28-ESR (ρ = −0.11, *p*<0.01), SDAI (ρ = −0.16, *p*<0.001), MMP-3 (ρ = −0.21, *p*<0.0001), and HAQ level (ρ = −0.13, *p*<0.01). These results suggested that higher intake frequency of vegetables was associated with lower disease activity. Considering the possibility of confounders which influences of the results, further analysis was made as follows.

**Table 3:** Correlation between food intake frequency and disease activity.

Food	DAS28-ESR	SDAI	MMP-3	HAQ
Meat	0.045	0.029	-0.14*	-0.057
Fish	0.086	0.0033	-0.018	-0.033
Tofu	0.018	-0.019	-0.033	-0.017
Egg	-0.025	-0.074	-0.010	-0.071
Milk	0.0060	-0.041	-0.020	-0.060
Vegetable	-0.11*	-0.16***	-0.21****	-0.13**
Fruits	0.063	-0.030	-0.062	-0.027
Fried food	-0.045	-0.035	0.031	-0.13**
Cake	0.046	-0.014	-0.090	-0.023
Juice	0.074	0.077	0.10*	-0.064
Snack food	-0.0028	0.037	0.048	-0.047
Sweets	-0.011	-0.026	-0.019	-0.11*
Miso soup	0.0056	-0.0085	-0.083	-0.086
Pickles	0.047	-0.0095	0.025	0.015
Ham	0.0042	-0.030	0.026	-0.057
Frozen food	-0.017	-0.027	0.097*	-0.077
Small fish	0.11*	0.017	0.048	0.14**
Canned tuna	-0.071	-0.080	-0.033	-0.13**
Squid	-0.054	0.064	0.016	-0.040
Shellfish	-0.072	-0.094*	0.029	-0.040
Roe	-0.038	-0.065	-0.013	-0.057
Fish paste	0.070	0.0006	0.15**	0.035

Values are expressed as Spearman’s rank correlation coefficients.

* *p*<0.05.

**p<0.01.

***p<0.001.

****p<0.0001.

### Identification of food patterns by factor analysis

The correlations among 22 food items were too complex to make further analysis (data not shown), which required simplifying the relations among the 22 food items. In order to categorize the 22 food items into several food patterns, factor analysis with varimax rotation was done. The analysis showed that the 22 food items were able to be categorized into five factors. Based on the food character of each component, we made the nomenclatures as follows: “seafoods”, “vegetables/fruits”, “meats/fried foods”, “snacks”, and “processed foods” (Table 4).

**Table 4:** Identification of food patterns by factor analysis.

Foods	Factor 1 “seafoods”	Factor 2 “vegetables /fruits”	Factor 3 “meats/fried foods”	Factor 4 “snacks”	Factor 5 “processed foods”
Shellfish	**0.70**	0.14	0.10	0.080	0.036
Squid	**0.65**	0.026	0.15	0.17	0.095
Roe	**0.55**	-0.076	0.013	0.18	0.17
Small fish	**0.48**	0.33	0.035	-0.046	-0.0075
Pickles	**0.44**	0.099	0.075	0.028	0.14
Canned tuna	0.25	0.14	0.13	0.16	0.18
Fruits	0.22	**0.70**	0.090	-0.046	-0.057
Vegetable	0.069	**0.63**	0.22	-0.16	-0.012
Milk	-0.020	**0.45**	0.087	0.053	0.068
Cake	0.13	0.38	0.23	0.27	0.0032
Meat	-0.018	0.14	**0.66**	0.14	0.069
Fried food	0.17	0.035	**0.53**	0.34	0.17
Egg	0.058	0.28	**0.51**	0.031	0.23
Fish	**0.41**	0.32	**0.49**	0.087	-0.076
Tofu	0.22	**0.43**	**0.46**	0.012	-0.013
Miso soup	0.26	0.27	0.29	0.032	0.074
Snack food	0.015	-0.033	0.062	**0.76**	0.082
Sweets	0.13	0.062	0.068	**0.46**	-0.024
Juice	0.12	-0.029	0.083	0.39	0.18
Ham	0.26	0.077	0.22	0.097	**0.74**
Fish paste	**0.42**	0.045	-0.018	0.16	**0.45**
Frozen food	0.057	-0.17	0.13	0.34	0.34

Values are expressed as correlation coefficients between foods and five factors identified by factor analysis.

Bold values are >0.40.

### Correlations between the five food factors and disease activity

To show the correlation among the five food factors and disease activity, multiple regression analysis was done, adjusted for the covariates which were selected by clinical significance, including age, sex, disease duration, Body Mass Index (BMI), drugs (methotrexate, prednisolone, and biologics), RF and anti-CCP antibody titers (Table 5). There was no multicollinearity among these covariates (data not shown). “Seafoods” had statistically significant negative correlations with DAS28-ESR (β = −0.15, *p*<0.01), SDAI (β = −0.18, *p*<0.001), MMP-3 levels (β = −0.15, *p*<0.01), and HAQ (β = −0.24, *p*<0.0001). “Vegetables/fruits” had statistically significant negative correlations with SDAI (β = −0.11 *p*<0.05), MMP-3 level (β = −0.12, *p*<0.01), and HAQ (β = −0.11, *p*<0.05). These results suggested that high intake frequency of vegetables and/or seafoods was correlated with low disease activity.

**Table 5:** Multivariate analysis for correlation between the five food patterns and RA disease activity.

Factors	DAS28-ESR	SDAI	MMP-3	HAQ
Age	0.32****	0.16**	0.25****	0.35****
Disease duration	0.13**	0.15**	0.033	0.23****
Sex (1 man, 0 women)	-0.19****	-0.038	0.17***	-0.11*
BMI	-0.07	-0.069	-0.08	-0.017
Methotrexate quantity	0.067	0.034	-0.044	-0.09*
Prednisolone quantity	0.15**	0.2****	0.3****	0.14**
Biologics use	-0.1*	-0.025	0.0061	0.035
RF titer	0.2****	0.16**	0.068	0.14**
ACPA titer	0.071	0.038	0.039	0.017
Factor 1 “seafood”	-0.089	-0.11*	-0.12**	-0.11*
Factor 2 “vegetables/fruits”	-0.15**	-0.18***	-0.15**	-0.24****
Factor 3 “meat/fried foods”	0.032	0.07	-0.0036	0.0088
Factor 4 “snacks”	0.021	0.00017	-0.046	-0.037
Factor 5 “processed foods”	0.057	0.052	0.11*	-0.038

Results are expressed as standardized beta coefficients.

ACPA: anti-cyclic citrullinated peptide antibody, RF: rheumatoid factor.

* *p*<0.05.

**p<0.01.

***p<0.001.

****p<0.0001.

## Discussion

In this cross-sectional retrospective study, the dietary habits detected with 22-item food frequency questionnaire (FFQ) were collected from 441 RA patients, and their correlations with RA disease activity were statistically analyzed. This cohort was characterized by long disease duration, low disease activity and high frequency of DMARD use (Table 1). The univariate analysis showed that the food intake frequency of vegetables negatively correlated with RA disease activity. The factor analysis with varimax rotation showed that in this study the 22 food items could be categorized into five dietary patterns, as follows: “seafoods”, “vegetables/fruits”, “meats/fried foods”, “snacks”, and “processed foods” (Table 4). Using these factors, multivariate analysis adjusted for clinically significant confounders showed negative correlation of “seafoods” and “vegetables/fruits” with disease activity markers (Table 5).

The demographics of this cohort suggested that most of the patients might have stable disease activities and experience few recent therapeutics changes. In the daily clinical practice, disease activity of RA in early stage is mainly influenced by the use of DMARDs, including methotrexate, biologics and oral corticosteroid. This leads to the difficulty in detecting the impact of dietary habits on disease activity of RA which is probably milder than that of DMARDs. In this cohort, the small impact of therapy on disease activity might lead to the desirable conditions for evaluating the impact of dietary habits.

In previous reports from Western countries, high consumption of fishes containing omega-3 PUFAs was related to low RA disease activity, whereas greater consumption of fats and sugars was related to high RA disease activity [4]. In another report, it was suggested that high intake of fish might be associated with low disease activity [21]. The findings in this study are consistent with the findings in these previous reports.

In previous reports from Japan, intake of omega3-PUFAs, fish oil and monounsaturated fatty acids might be associated with low disease activity [11, 12]. The findings of these reports are consistent with the findings of this study, while the numbers of patients in these reports were smaller than the numbers in this study.

There are several strong points in this study. First, the number of patients in this study was larger than that in previous reports, because the FFQ in this study was more concise than the other well-known FFQs. Second, the five dietary patterns were identified by the factor analysis. The multivariate analysis with these patterns revealed the negative correlations of the “vegetables/fruits” or “seafoods” with disease activity. The negative correlation of “seafoods” with disease activity had not been detected by the univariate analysis with the individual food items. In the factor analysis, “Meats/fried foods” had a strong correlation with fish. This implies that in the univariate analysis the positive correlation of fish with disease activity might be neutralized by meat or fried foods. From this point of view, factor analysis might be useful to elucidate the genuine impact of dietary habits on disease activity.

These results allow two possible interpretations. First, greater consumption of vegetables and/or seafoods decreases RA disease activity. In previous studies, the Mediterranean diet, which is rich in vegetables, alleviated arthralgia in RA patients [6]. In mice, dietary fiber increased the number of regulatory T cells by influencing intestinal microbiota [22], which might diminish the chronic arthritis of RA. In epidemiological and basic studies [6, 21, 23], omega-3 polyunsaturated fatty acids (PUFAs) and their metabolites, which are rich in seafoods, decreased the severity of arthritis. Protein may play a beneficial role in rheumatoid arthritis. Chronic inflammation of RA leads to sarcopenia, which might be improved by the protein from seafoods[24]. Second, disease activity might influence on dietary habits. For example, high disease activity of RA might decrease the capacity to cook meals, leading to diminished frequency of vegetable or seafood consumption [25]. Prospective interventional studies are necessary to elucidate the causality.

There are several limitations in our study. First, this retrospective study cannot determine the causality or rule out the possibility of unmeasured confounders, such as socioeconomic status. In this study, the high use rate of biological DMARDs implies high-income population. This might lead to small impact of socioeconomic status on disease activity or dietary habits. Prospective interventional studies are needed. Second, this study might be affected by recall bias because FFQ was took to measure dietary habits. The difference between actual and recalled dietary habits may be caused by inaccurate remembrance of the past dietary habits [19]. Total intake amount of food is also important. However, the difficulty in estimation of intake amount of food by patients may lead to the inaccurate answer. In contrast, the FFQ in this study is easy to answer, especially for those who does not cook for themselves. Therefore, the FFQ might be less affected by recall bias than questionnaire on the amount.

It might be necessary to combine FFQ with other methods, such as 24-hour recall method for measuring dietary habits. Third, the reproducibility of this FFQ has not yet been confirmed. Most of the well-known FFQ are too long to use in real clinical situations [26]. The 22-items FFQ in this study is concise enough to be took in daily practice. Fourth, because the patients in this study had long standing and mild disease, this result might not be applicable to other populations. The same evaluation in a different RA population is necessary. Fifth, “seafoods” or “vegetables/fruits” includes various foods. Detailed evaluation of vegetables or fish is remained to elucidate which kind of food stuffs have an association with disease activity. Generally, extremely unbalanced diet easily causes unexpected weight loss and malnutrition especially in chronic inflammatory diseases. Needless to say, balanced diet is important to maintain good health.

In conclusion, this study revealed that high frequency of vegetables and/or seafoods was correlated with low disease activity in this Japanese cohort. To our best knowledge, few reports from the non-western countries have focused on a comprehensive evaluation of dietary habits and their relationship to disease activity. Our study suggests that more intake of vegetables or seafoods could reduce RA disease activity. Further study is needed to clarify the precise impact of dietary habits on disease activity of RA.

## Supporting information

S1 File Detailed study data. (XLSX)
